# Novel low-cost approach to the treatment of ocular surface squamous neoplasia using pattern scanning laser photocoagulation

**DOI:** 10.5935/0004-2749.20200094

**Published:** 2024-02-11

**Authors:** Ever Ernesto Caso Rodriguez, Karlos Frederico Castelo Branco Sancho, Patricia Mencaroni Kange, Jeison de Nadai Barros, Aline Sutili Toledo, Melina Morales, Rubens Belfort Neto

**Affiliations:** 1 Ophthalmology and Visual Sciences Department, Universidade Federal de São Paulo, Brazil; 2 Department of Ophthalmology, Hospital das Clínicas, Faculdade de Medicina da Universidade de São Paulo, São Paulo, SP, Brazil

**Keywords:** Carcinoma, squamous cell/diagnosis, Conjunctival neoplasms/therapy, Laser therapy, Photocoagulation, Carcinoma de células escamosas/diagnóstico, Neoplasia da túnica conjuntiva/terapia, Terapia a laser, Fotocoagulação

## Abstract

**Purpose:**

To evaluate the safety and 12-month effect of treatment with pattern scanning laser photocoagulation for ocular surface squamous neoplasia in a low-resource setting with extremely limited access to an operating room.

**Methods:**

Adult patients with a clinical diagnosis of ocular surface squamous neoplasia underwent a complete ophthalmologic examination. After topical anesthesia and instillation of toluidine blue 1%, the lesion was treated using pattern scanning photocoagulation for a duration time that varied from 20 to 100 ms and power from 600 to 1,800 mW. Patients were examined on a weekly basis for the first month and underwent weekly retreatment of the remaining lesions, as necessary. Patients had a minimum follow-up of 12 months.

**Results:**

Thirty-eight patients (38 eyes) were included. All patients had clinical ocular surface squamous neoplasia that was confirmed by impression cytology. The age of patients ranged from 40 to 83 years (average: 65.5 years) and 28 of them were males (74%). The patients were divided into two groups: group I (immunocompetent) and group II (immunosuppressed). In group I, 23 patients (74%) presented complete response with lesion control after laser treatment alone. In group II, two of seven patients (28%) showed treatment response during the follow-up. The average number of treatments was 2.5 (one to six laser treatments). Procedures were well tolerated.

**Conclusion:**

Short-term results of the laser photocoagulation approach for the treatment of ocular surface squamous neoplasia conjunctival lesions were favorable, with a 74% success rate observed in immunocompetent patients. This novel strategy is a less resource-intensive alternative that could demonstrate its usefulness in settings with shortages in operating rooms and in recurrent cases. Studies with longer follow-ups and larger sample sizes are warranted to confirm our findings and evaluate the effectiveness of laser treatment in association with topical chemotherapy.

## Abstract (pt)

**Objetivo:**

Avaliar a segurança e o efeito de 12 meses de tratamento com fotocoagulação pelo pattern scanning laser para neoplasia escamosa da superfície ocular em um ambiente com poucos recursos e acesso extremamente limitado a um tratamento cirúrgico.

**Métodos:**

Pacientes adultos com diagnóstico de neoplasia escamosa de superfície ocular foram submetidos a exame oftalmológico completo. Após anestesia tópica e instilação de azul de toluidina 1%, a lesão foi tratada com laser por um tempo de duração que variou de 20 a 100 ms e potência de 600 a 1800 mW. Os pacientes foram examinados semanalmente durante o primeiro mês e foram retratados semanalmente das lesões restantes, conforme necessário. Os pacientes tiveram um seguimento mínimo de 12 meses.

**Resultados:**

Trinta e oito pacientes (38 olhos) foram incluídos no estudo. Todos os pacientes apresentaram neoplasia escamosa da superfície ocular clínica, confirmada por citologia de impressão. A idade dos pacientes variou entre 40 e 83 anos (média de 65.5 anos) e 28 deles eram do sexo masculino (74%). Os pacientes foram divididos em dois grupos: Grupo I (imunocompetente) e grupo II (imunossuprimido). No grupo I, 23 pacientes (74%) apresentaram resposta completa com o controle da lesão após o tratamento com laser. No grupo II, dois dos sete pacientes (28%) apresentaram resposta ao tratamento durante o acompanhamento. A média de aplicações de laser foi de 2,5 (1 a 6 aplicações). Os procedimentos foram bem tolerados.

**Conclusões:**

Os resultados a curto prazo da abordagem de fotocoagulação a laser para o tratamento das lesões conjuntivais de neoplasia escamosa de superfície ocular foram favoráveis, com uma taxa de sucesso de 74% observada em pacientes imunocompetentes. Essa nova estratégia é uma alternativa menos intensiva em recursos que pode demonstrar sua utilidade em ambientes com escassez de salas cirúrgicas e em casos recorrentes. Estudos com acompanhamentos mais longos e amostras maiores são necessários para confirmar nossos achados e avaliar a eficácia do tratamento a laser associado à quimioterapia tópica.

## INTRODUCTION

The most common type of ocular cancer in adults is ocular surface squamous neoplasia (OSSN). This term encompasses dysplasia, carcinoma *in situ*, intraepithelial neoplasia, and squamous cell carcinoma (SCC) of the cornea and/or conjunctiva^(1)^. Risk factors include exposure to ultraviolet light B^(2^,^3)^; therefore, a higher incidence is observed in countries close to the equator^(4)^. Other risk factors associated with OSSN are fair skin, human papilloma virus infection^(5^,^6)^, human immunodeficiency virus^(7)^, other forms of immunosuppression^(8^-^13)^, age, male sex^(1^,^2^,^14)^, and xeroderma pigmentosum^(2^,^15)^.

Current treatments for OSSN include topical chemotherapy with interferon-α2b, mitomycin C, and 5-fluorouracil as a single treatment. Surgery with a “no touch” technique is used during excision of OSSN, with wide surgical margins of 2-3 mm and use of cryotherapy at the edges of the lesion; surgery with adjunctive antimetabolites is another popular option^(1^,^4)^.

In dermatology, the treatment of non-melanoma skin cancer with lasers causes light absorption by blood vessels in the targeted area, resulting in thermal distraction that subsequently leads to tumor regression^(16^,^17)^. This targeted vascular photothermal destruction preserves the normal surrounding area and can lead to excellent cosmesis^(1^,^2^,^17^,^18^-^21)^. There are four major laser types used in the treatment of skin cancers: solid-state, diode, dye, and gas^(22)^. Several studies reported results for the treatment of basal cell carcinoma: pulse dye lasers have shown promising results with a complete clinical response in up to 95% of patients. In addition, the CO_2_ laser exhibits high efficiency (85-100% cure), excellent cosmetic outcomes, and minimal complications for the treatment of basal cell carcinoma^(18)^. There are few studies testing the use of laser treatment for SCC. In the largest study, 44 of 48 patients with SCC were treated with the CO_2_ laser; they had a total clearance rate of 97.7% and a recurrence rate of 6.8% on average after 18 months of follow-up^(23)^.

Patients were treated using pattern scan laser photocoagulation without recurrence after 6 months in both instances.

An internal review of referral patterns shows that nearly 35% of new referrals to our hospital center in São Paulo are for epithelial lesions, including papillomas, dysplasia, and SCC. The large volume of surgical cases represents a serious challenge to our ocular oncology service. In a low-resource setting, such as the Brazilian public health system, shortage in operating room time is very common. Moreover, the associated direct patient costs for topical chemotherapeutic agents, such as mitomycin C and interferon therapy, render these treatments inaccessible for most uninsured patients. Even if these drugs become available on a compassion basis, patients face difficulties, including the transport of interferon from the pharmacy to their home, its storage at a constant 4°C, and its replacement every 15 days for 3-6 months.

Although expensive, the green diode laser is readily available because it is used to treat retinal diseases, including diabetic retinopathy. Our team sought to develop a new pattern scanning laser photocoagulation-based treatment platform for OSSN, aiming to determine its safety and efficacy.

## METHODS

Adult patients clinically diagnosed with OSSN at the Ophthalmology Department of the São Paulo Hospital (São Paulo, Brazil) were invited to participate in the study. The study was reviewed and approved by the appropriate ethics review board (clinical trial registration number: 65397016.8.0000.5505). On the day of initial laser treatment, patients underwent a complete ophthalmologic examination, including anterior segment photography and impression cytology. Signed informed consent was provided by all patients.

Patients received topical anesthesia with proparacaine, followed by subconjunctival injection with 0.20.5 ml xylocaine in patients who complained of pain. Subsequently, instillation of one drop of toluidine blue 1% was used to increase laser absorption, followed by treatment with a 532-nm diode-pumped solid stage laser (PASCAL^®^ Streamline; Topcon Medical Laser Systems, Santa Clara, CA, USA). The duration time was 20-100 ms, the laser power varied (600-1,800 mW), the spot size was 200 µm, and either a single or pattern photocoagulation with a spacing of 200-µm shooting was used. Between 300 and 1,400 laser shots were delivered, depending on the discomfort threshold of the patient and lesion size. Initial settings were power of 600 mW, spot size of 200 µm, and duration of 100 ms. Power was increased to provide whitening of the lesion surface upon laser therapy. The entire lesion was treated with confluent marks. A cotton swab was used in pedunculated lesions to allow lasering of the lateral and posterior margins. Different scanning patterns were used, based on the lesion size. The healthy appearing surrounding 2 mm of the conjunctiva was also treated. Topical medications were not prescribed post procedure and bleeding was not observed. Staff present in the room wore surgical masks for protection against aerosolized virus particles. Patients were examined on a weekly basis via slit-lamp examination and anterior segment photography for the first month; photocoagulative retreatment was performed for persistent lesions. After the clinical resolution of the lesion, patients were examined in follow-up visits after 1, 2, and 4 months, and every 3 months thereafter.

Growth of the lesion despite laser therapy, absence of improvement after three therapy sessions, and lesion recurrence despite initial response indicated failure of treatment. In these cases, patients were treated with excisional biopsy, topical chemotherapy, or subconjunctival injection of interferon (3,000,000 IU/0.5 ml).

## RESULTS

We report results for 38 patients (38 eyes) that met the inclusion criteria of the study, and were followed up for ≥12 months after receiving treatment between Octo ber 2016 to May 2018 at the São Paulo Hospital. All patients had clinical OSSN confirmed through impression cytology and had at least two imprints obtained over the same area of the lesion. During laser therapy, patients experienced some mild discomfort, which persisted for 1-2 days after treatment and was well tolerated. There were no other side effects reported. A few patients developed mild discoria below the treated area and the technique was modified, aiming the laser to the limbus to limit photoexposure of the iris. Topical medications were not prescribed post procedure.

The age of patients ranged 40-83 years (average: 65.5 years), and 28 patients were males (74%). Seven patients were immunocompromized due to organ transplantation (i.e., kidney transplantation) and immunosuppression for autoimmune diseases. The drugs used were azathioprine and cyclosporine.

**Figure 1:**
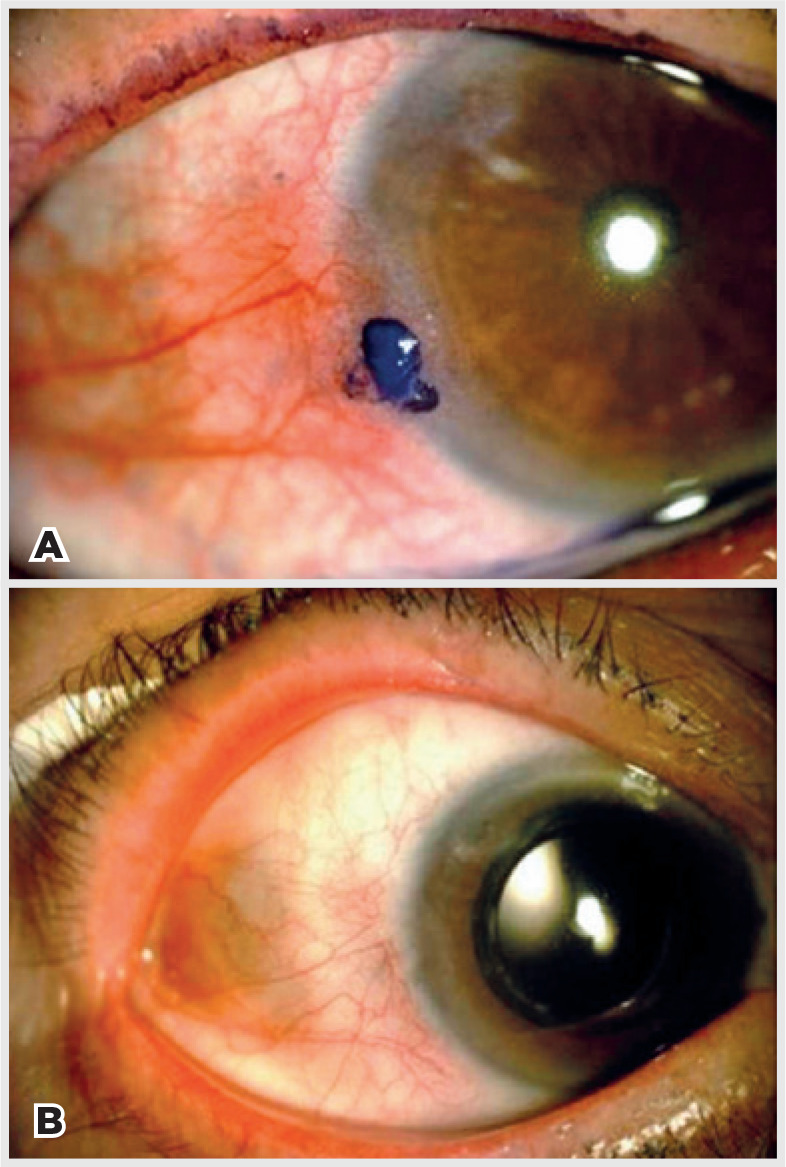
A pre-treatment color photograph of patient 1 with OSSN evidenced by toluidine blue 1%. A) and Stage T2 by AJCC^(24)^. B) complete regression at 15-month follow-up.

**Figure 2:**
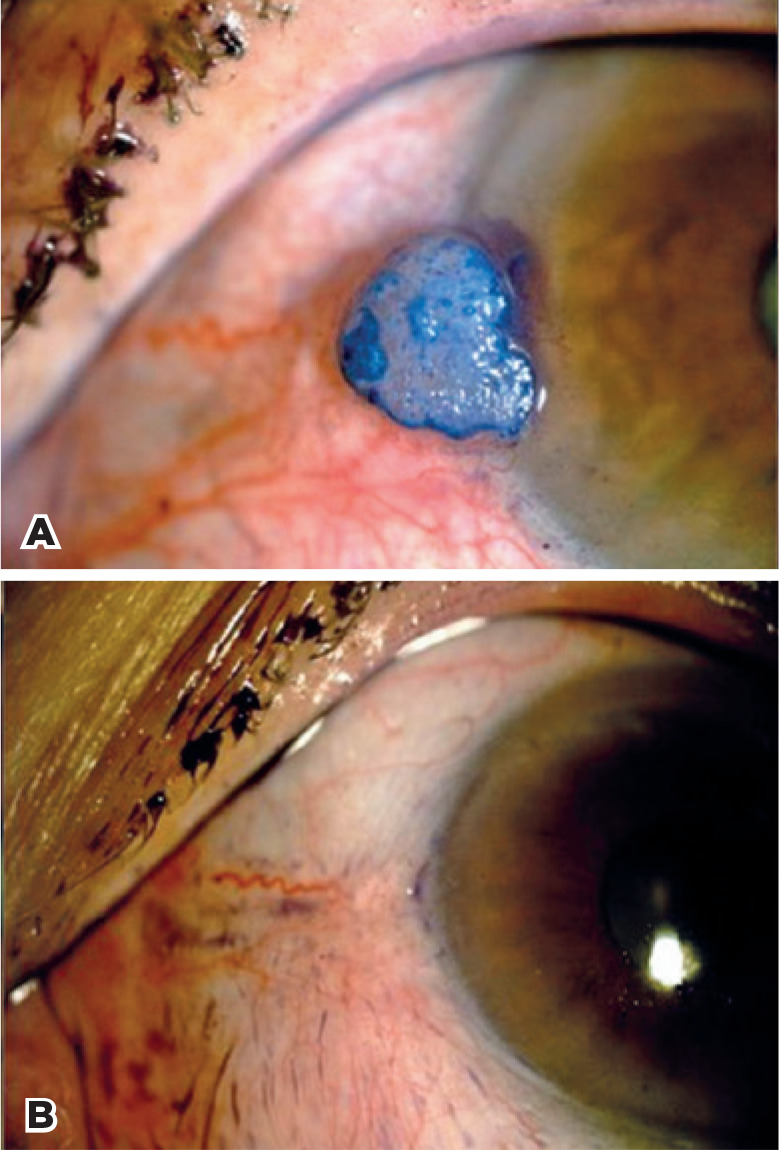
A pre-treatment color photograph of patient 2 with OSSN of the conjunctiva with involvement of the cornea. A) and Stage T1 by AJCC^(24)^. B) complete regression at 13-month follow-up.

**Figure 3:**
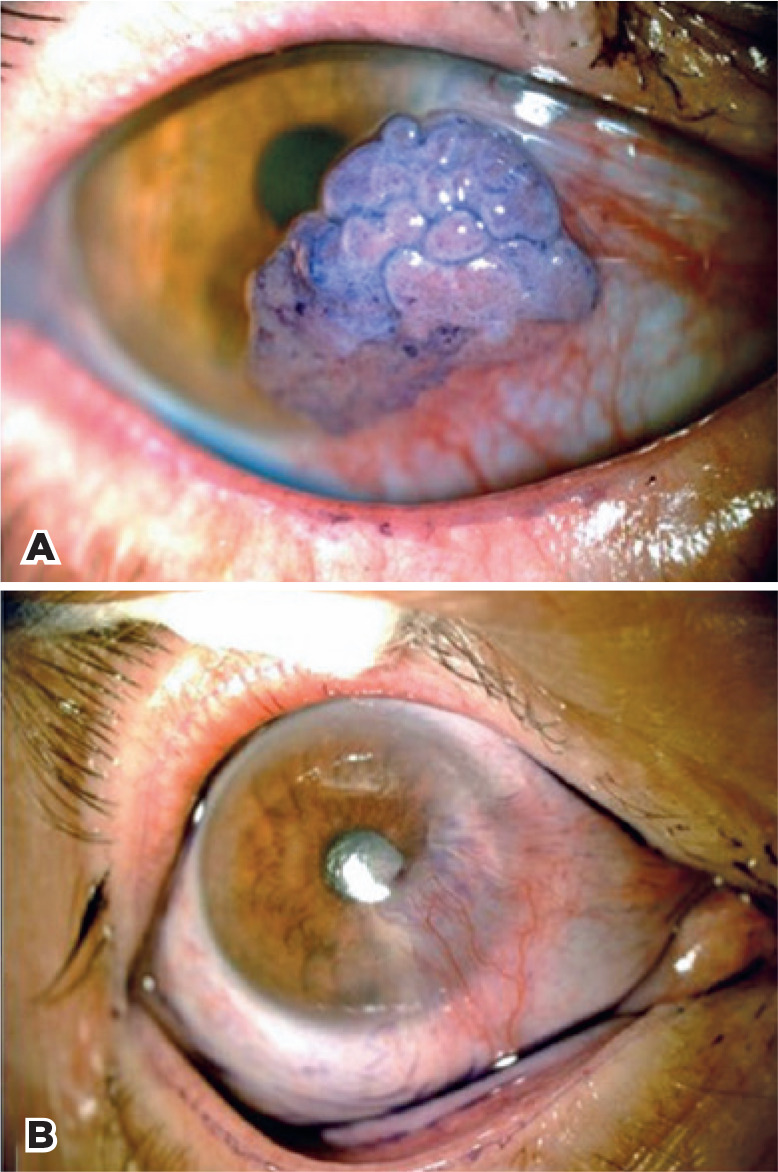
A pre-treatment color photograph of patient 3 with OSSN of the conjunctiva with involvement of the cornea. A) and Stage T2 by AJCC ^(24)^. B) com plete regression of the neoplasia and residual pannus at 20-month follow-up.

**Figure 4:**
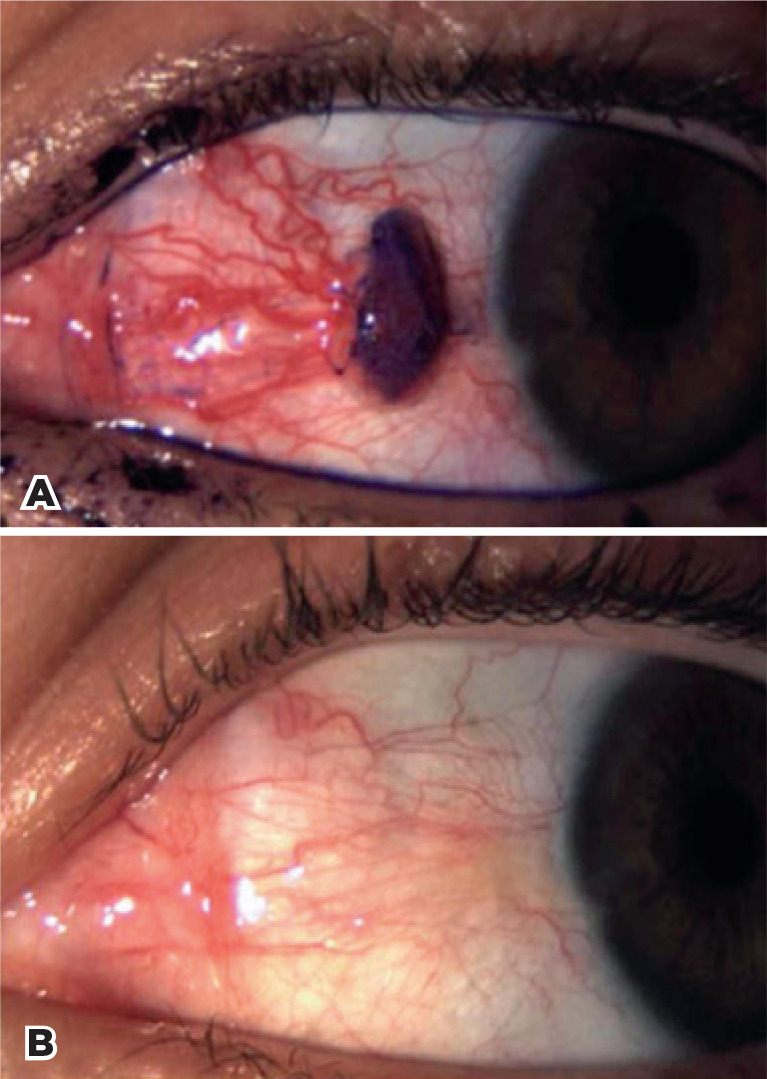
A pre-treatment color photograph of patient 4 (at 3 months of pregnancy) with OSSN of the conjunctiva. A) and Stage Tis by AJCC^(24)^. B) complete regression at 12-month follow-up.

The patients were divided into two groups: group I (immunocompetent) and group II (immunosuppressed). In group I, 23 patients (74%) presented complete response with lesion control after laser treatment alone. In group II, two of seven immunosuppressed patients (28%) exhibited treatment response during the follow-up. Both groups had lesions between 1 and 3 clock hours of limbus. The average number of treatments was 2.5 (one to six laser treatments). Procedures were well tolerated, with minor adverse effects, including mild discomfort up to 2 days after treatment. Figures 1-4 illustrate the preand post-treatment results.

OSSN= ocular surface squamous neoplasia; AJCC= American Joint Committee on Cancer.

OSSN= ocular surface squamous neoplasia; AJCC= American Joint Committee on Cancer.

OSSN= ocular surface squamous neoplasia; AJCC= American Joint Committee on Cancer.

OSSN= ocular surface squamous neoplasia; AJCC= American Joint Committee on Cancer.

## DISCUSSION

This proof of concept study showed that a pattern scanning laser approach with 1% toluidine blue is a safe and effective minimally invasive treatment for OSSN lesions in the short term. There were no significant complications recorded; patients experienced some mild discomfort and conjunctival hyperemia, which persisted for 1-2 days after treatment and were well tolerated. After observing mild discoria in some patients, we aimed the laser toward the limbus to minimize absorption by the iris pigment. A significant number of lesions demonstrated complete regression following one to six treatment sessions, with the mildest of discomfort reported within the first 48 h. The use of three galvanometers to create larger scanning patterns in this specific laser proved to be faster and less cumbersome for these patients, rendering the treatment more effective.

The novel use of toluidine blue 1% in this protocol is appropriate. Considering its long history of ophthalmic use and that it has been shown to stain areas of high mitotic activity, it is invaluable for observing separate areas of early neoplastic or dysplastic epithelial growth and establishing therapy margins^(25)^. We expected the blue dye to more efficiently absorb the laser energy, thereby increasing the effectiveness and specificity of the treatment. It is currently unclear whether alterations in the concentration or exposure time affect the energy required or the recurrence rate. Moreover, toluidine blue at concentrations ranging 0.05-1% is a safe compound for use on the ocular surface and oral cavity without significant adverse effects^(26^-^28)^.

Treatment was more successful for raised lesions than flat ones. Lesions with a mainly corneal component and extensive exhibited worse response. This finding suggests that the treatment is more effective in treating carcinomas than epithelial dysplasia. Our group has reported a 100% success rate in treating papillomas of the conjunctiva using the therapeutic modality reported in this article. Despite being benign lesions, the papillomas were larger compared with dysplasias^(26)^.

Immunosuppressed patients present a challenge for the control of initial disease. Because of a higher chance of frequent recurrences, these patients may require multiple surgical interventions. In this group of patients, use of a laser as a single treatment was ineffective. However, it may be useful in association with topical chemotherapy or immunomodulation with topical interferon.

The lower effectiveness of laser treatment observed in group II (immunosuppressed) may be explained by the higher incidence of OSSN (20-fold increase) in patients with kidney transplantation using azathioprine and cyclosporine^(12)^, and prolonged use of immunosuppressive treatment. A systematic review and meta-analysis found an increased risk of cutaneous SCC in recipients of organ transplants treated with azathioprine^(8)^. Mutagenic effects have been observed following cellular exposure to the combination of ultraviolet light and azathioprine (azathioprine causing the accumulation of 6-thioguanine in DNA). Of note, 6-thioguanine and ultraviolet light are synergistically mutagenic^(8^,^10)^. Moreover, changes in cell morphology and inhibition of DNA repair, apoptosis, and p53 function have been associated with exposure to cyclosporine^(9^,^13)^.

These results are comparable with the current standard of care, surgical excision, and cryotherapy with or without adjuvant topical chemotherapy. However, this approach added the benefit of increased access to care, decreased post-operative dependence on medication, and cost savings both for patients and payers.

OSSN can occur in all age groups; it is most commonly reported in men in their 60s-70s who live close to the equator^(1^,^2)^. The average age in this study was 65.5 years, which is congruent with the average age of the conjunctival carcinoma population. In addition, the ratio of males to females in this study was 5:2 (i.e., 74% male predominance). Thus, this study exhibited concordance with the literature in terms of the most affected age. The effectiveness of treatment was higher in group I (immunocompetent) (74% complete response) and less effectiveness in group II (24% complete response).

This study had several limitations. Firstly, the small sample (38 patients) is clearly insufficient to draw conclusions regarding changes in practice. Nevertheless, we have demonstrated the initial efficacy of this technique and its potential in treating OSSN lesions, such as SCC. Secondly, a 1-year follow-up is considered a short time to assess the possible long-term complications after laser use and recurrence of OSSN. Further studies with longer follow-up periods are warranted to address these topics. Thirdly, this study lacked a histopathological analysis. However, it was considered pointless to perform an incisional biopsy merely for the confirmation of the clinical diagnosis without excising the entire lesion.

Impression cytology (IC) has been shown to be extremely reliable in the diagnosis of OSSN^(20^,^29)^. Although IC cannot replace histology, it plays an important role in the diagnosis and management of patients with OSSN in a less invasive manner^(20)^. This method has both advantages and limitations. The advantages are as follows: (a) it provides a source of intact and well-preserved epithelial cells from the ocular surface in any type of ocular surface pathology; (b) it is a nonsurgical, easy-to-perform, and inexpensive technique that can always be performed on an outpatient basis; and (c) repeated IC sampling in the same patient over time is an excellent approach to demonstrate changes caused by a certain event, monitor the progress of a disease, or follow the effect of a therapeutic intervention. The limitations of this technique are as follows. Firstly, the IC analysis accesses only superficial layers of cells, which are not representative of the deeper layers^(20)^. Thus, sensibility is increased by the repeated collection of multiple samples^(19^,^20)^, as performed in the present study. All patients had at least two imprints obtained over the same area of the lesion. Secondly, the cytological profile of the cells obtained in the IC specimen is not representative of the entire sample as observed in the histology. Finally, this method is unable to reliably detect invasion^(20)^.

It is imperative to continue evaluating this new treatment using different laser and light sources and staying substances, as well as its association with other forms of treatment (e.g., reducing tumor volume prior to use of topical chemotherapy or treating small conjunctival recurrences after surgical or topical treatment). Overall, this proof of concept study demonstrates good short-term efficacy of this modality with retreatment and minimal side effects. Furthermore, there were no serious complications related to treatment of OSSN with pattern laser with toluidine blue, and excellent cosmetic outcomes were observed. The association of laser and topical medication is another therapeutic option that should be evaluated.

This technique is more cost effective than the current standard of care and more accessible to those in the developing world or settings with limited operating room availability. Although a photocoagulation laser is an expensive piece of equipment, it is widely available in most centers, owing to its wide use for the treatment of diabetic retinopathy and other retinal diseases.
